# Judicial demands for prostate cancer treatment in Brazil: androgen receptor pathway inhibitors are an urgent public health problem

**DOI:** 10.31744/einstein_journal/2025GS1301

**Published:** 2025-09-11

**Authors:** Mariana Avelar da Silveira, Carla de Oliveira Ricomini, Reuli Cordeiro da Silva, Flavio Tocci Moreira, Carlos Henrique Sartorato Pedrotti, Vanessa Damazio Teich, Luciana Holtz de Camargo Barros, Sidney Glina, Fernando Korkes

**Affiliations:** 1 Hospital Israelita Albert Einstein São Paulo SP Brazil Hospital Israelita Albert Einstein, São Paulo, SP, Brazil.; 2 Centro Universitário FMABC Santo André SP Brazil Centro Universitário FMABC, Santo André, SP, Brazil.; 3 Instituto Oncoguia São Paulo SP Brazil Instituto Oncoguia, São Paulo, SP, Brazil.

**Keywords:** Prostate neoplasms, Judicial role, Receptors, androgen, Public helath, Publoic policy, Cost analysis, Brazil

## Abstract

**Objective:**

Considering the difficulties of Brazil's Public Health System in providing comprehensive care, the present article aimed to assess legal cases filed against the Brazilian Public Health System in pursuit of finer treatment for patients with prostate cancer. In doing so, this study aims to provide the intel necessary to support new public health policies, as well as to assess the financial impact on the Federation, thereby accounting for better outcomes in patient care.

**Methods:**

This cross-sectional study evaluated the technical notes issued by the Technical Support Centers of the Judiciary regarding lawsuits from patients against the Brazilian Public Health System from 2019 to 2024, concerning prostate cancer to better advise the formulation of public policies regarding oncologic care.

**Results:**

A total of 3,136 technical notes were issued. Lawsuits were intended to obtain either medication (88.6%), procedures (10.2%), or other healthcare products (1.2%). Most medications requested were Androgen Receptor Pathway Inhibitors in 2,470 cases, corresponding to approximately 88.9% of the demand for drugs and 78.8% of all treatment demands. Overall, abiraterone was the most requested intervention, accounting for 51.2% of the technical notes issued. As for the distribution of legal proceedings within Brazilian states, demands were more common in the Southern (42.2%), Northeastern (31.3%), Southeastern (14.1%), Central-western (11.5%), and Northern (0.86%) regions of Brazil. However, the Southeastern and Southern regions tend to pursue more expensive drugs.

**Conclusion:**

Therefore, there is a pressing need for strategic interventions to address the escalating healthcare litigation crisis in Brazil. Collaboration among government entities, the scientific community, the judiciary, and patient advocacy groups is essential for effective formulation.

## Highlights

■A total of 89% of the 3,136 legal demands sought medication.

■Androgen inhibitors amounted to 78.8% of all lawsuits, particularly abiraterone.

■There is a growth tendency in judicialization, with a 30-fold increase over 4 years.

■The estimated annual financial burden of judicialization on the Brazilian United Public Health System has reached BRL 783 million.

## In Brief

This cross-sectional study evaluated lawsuits against The Brazilian United Public Health System, seeking prostate cancer treatments to determine the impact of judicialization. Out of the 3,136 demands, 2,778 targeted medications, of which 78.8% were androgen inhibitors, particularly abiraterone. With a 30-fold increase in legal actions between 2019 and 2023, the projected costs reached BRL 783M/year, with unequal distribution throughout Brazil.

## Graphical abstract

**Figure:**
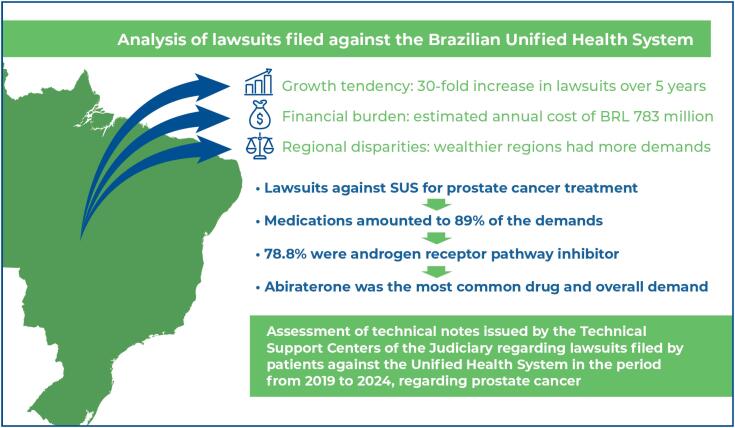

## INTRODUCTION

The Brazilian Public Health System (SUS - *Sistema Único de Saúde*) was established in 1990, through Law No. 8080, which states that health is a fundamental right, the State being responsible for providing the indispensable conditions for its circumspect fruition.^(1)^

To fulfill this constitutional duty, the Brazilian SUS strives to yield a wide variety of medical resources whose cohesive and interdependent functioning is essential for the proper integral and updated healthcare of those in need.^(2)^

In terms of oncological care, considering the time sensitivity associated with the disease, the effective performance of the aforementioned resources is the key to guaranteeing the success of cancer treatment. However, swift changes in oncological recommendations and protocols for cancer treatment can be difficult to match, especially considering the whopping magnitude of Brazil's population and territory, apart from its still developing economic and social disparities.

Such failures are clear disregard for a constitutional right, as well as quite catastrophic in certain cases. To mitigate the shortcomings of this scenario, a common strategy has been petition for access to certain treatments via the Brazilian judiciary system, thus assuring patients of their legal rights.^(3)^

The pursuit of legal action has become relevant in recent years and has even begun to influence national drug policy, culminating in the creation of the Technical Support Centers of the Judiciary system (NATJUS - *Núcleo de Apoio Técnico do Judiciário*). The said Agency was developed to facilitate the search for legal aid to grant the desired access to health treatments. Since 2019, the NATJUS has issued over 50,000 technical notes, documents prepared through cooperation between the judiciary system and the relevant healthcare institutions responsible for providing judges with scientific expertise on the potential benefits of a particular technology for individual health treatment, addressing individual legal demands for medications (77%), procedures (18%), and health products (5%).^(4,5)^

In practical terms, one of the main reasons behind legal action pursued against Brazilian SUS regarding oncologic care is the discrepancy between the essential drugs determined by National List of Essential Medicines (RENAME - *Relação Nacional de Medicamentos Essenciais*), a list of pharmaceuticals that ought to be distributed by Brazilian SUS, and those approved by the National Committee for the Incorporation of New Technologies (CONITEC - *Comissão Nacional de Incorporação de Tecnologias no Sistema Único de Saúde*). The difficulties behind this delayed compliance can often be explained by the prohibitive cost of new medications, which imposes a high economic burden on Brazil's healthcare system.^(4)^

Despite being one of the leading strategies in current treatment of prostate cancer, Androgen Receptor Pathway Inhibitors are not widely incorporated into CONITEC. An exception to this scenario is abiraterone, which made its debut in the Brazilian SUS in 2019, specifically for the treatment of chemical castration-resistant metastatic prostate cancer. However, it has still remained unavailable in public settings.^(6-10)^

Understanding the reality of prostate cancer treatment is crucial for reforming public health policies, particularly considering the substantial financial burden associated with the treatment of these patients.^(11,12)^

## OBJECTIVE

The present study aimed to assess legal cases filed against the Brazilian Public Health System in pursuit of finer treatment for patients with prostate cancer in the hope of providing the information necessary to support new public health policies, thus leading to better health outcomes. The study also evaluates the financial impact on the Federation.

## METHODS

This cross-sectional study evaluated the technical notes corresponding to lawsuits filed against the SUS on prostate cancer treatment (ICD C61) from 2019 to 2023. Data from the judiciary (CNJ - *Conselho Nacional de Justiça*) were also obtained for further analysis.

These demands were issued by the Center for Technical Support of the Judiciary, a division of the Brazilian NATJUS, which aims to provide legal court opinions and technical responses grounded in scientific knowledge to assist and guide legal cases involving medical procedures and medication expenses.

As to the financial impact of judicialization, medication costs were determined based on the Chamber for the Regulation of the Pharmaceutical Market (CMED - *Câmara de Regulação do Mercado de Medicamentos*) list of the Health Ministry, factoring in an average 18% tax on government sales price (PMVG).^(13)^ For currency conversion, a rate based on Purchasing Power Parity (PPP) was applied using the value of USD 1 = BRL 2.64, according to data from the World Bank (2023).^(14)^

### Ethical considerations

Since only anonymous public institutions were considered for writing of this essay, Institutional Review Board approval was not necessary for this study, following National Health Council (CNS - *Conselho Nacional de Saúde*) Resolution No. 510/2016, Article 2.

## RESULTS

A total of 3,136 technical notes regarding legal actions involving patients undergoing treatment for prostate cancer were released. The plaintiffs were male patients with an average age of 70.41 ± 37.5 years (ranging from 25 to 100 years old). Lawsuits were filed for medications (n=2,778), medical procedures (n=322), and other healthcare products (n=36), as shown in table 1.

**Table 1:** Demands in 3,136 lawsuits notes evaluated and their outcomes

Reason		Requests	Favorable	Favorable %
Medication		2,778	1,600	57.6
	Bone therapy	25	21	84.0
	Chemotherapy	73	43	58.9
	Hormone therapy	121	47	38.8
	ARPIs	2,470	1,441	58.3
	Immunotherapy	3	1	33.3
	Targeted directed agents	12	8	66.7
	Teranostics	61	37	60.7
	Others	13	2	15.4
Procedure		322	230	71.4
	Complementary exams	106	77	72.6
	Radiotherapy	15	9	60.0
	Surgical procedures	105	79	75.2
	Others	96	65	677
Product		36	20	55.6

ARPIs: androgen receptor pathway inhibitors.

The most frequently requested medications included Androgen Receptor Pathway Inhibitors in 2,470 patients, which corresponded to approximately 88.9% of the demand for drugs and 78.8% of all treatment demands for patients with prostate cancer. The most commonly used pharmaceuticals were abiraterone (n=1,608; 65.1%), enzalutamide (n=606), and apalutamide (n=198). Overall, abiraterone was the most frequently requested intervention by patients with prostate cancer, accounting for 51.2% of all the technical notes issued. Additionally, patients were relatively successful with their judicial proceedings (Tables 1 and 2).

**Table 2:** Number of cases of lawsuits demanding drugs and their outcomes

Medication		Requests	Favorable	Favorable %
Bone therapy		25	21	84.0
	Zoledronic acid	19	16	84.2
	Denosumab	6	5	83.3
Chemotherapy		73	43	58.9
	Cabazitaxel	69	39	56.5
	Carboplatin	1	1	100.0
	Docetaxel	3	3	100.0
Hormone therapy		121	47	38.8
	Bicalutamide	18	6	33.3
	Cyproterone	19	2	10.5
	Degarelix	2	1	50.0
	Flutamide	5	4	80.0
	Goserelin	59	27	45.8
	Leuprorelin	15	6	40.0
	Triptorelin	3	1	33.3
ARPIs		2,470	1,441	58.3
	Abiraterone	1,608	1,107	68.8
	Apalutamide	198	97	49.0
	Darolutamide	58	19	32.8
	Enzalutamide	606	218	36.0
Immunotherapy		3	1	33.3
	Pembrolizumab	2	1	50.0
	Nivolumab	1	0	0.0
	Targeted agents	12	8	66.7
	Olaparib	12	8	66.7
Theranostics		61	37	60.7
	Lutetium 177	14	9	64.3
	223 Ra	47	28	59.6
Others		13	2	15.4

ARPIs: androgen receptor pathway inhibitors.

The other drug classes included traditional hormonal therapies (n=121), chemotherapy (n=73), and theranostics (n=61). While the first class had favorable recommendations roughly one-third of the time, the latter two had a better acceptance rate, close to 60% (Table 2).

Bone therapy (n=25), targeted agents (n=12), and immunotherapies (n=3) were the classes of medications that were rarely prescribed. As observed, among these, bone therapy was mostly granted and the most common drugs used were zoledronic acid (n=19), olaparib (n=12), and pembrolizumab (n=2).

During the assessed period, lawsuits with favorable requests seeking the most common demands amounted to 24.2 million USD (Table 3). If considered that 34.1% of all legal demands for medication had technical notes issued, this would project an annual expenditure of 70.9 million USD for the Federal Government. Most costly projected expenditures with demands for prostate cancer medications included abiraterone (44.6 million USD), enzalutamide (13.4 million USD), apalutamide (6.0 million USD), radium 223 (3.1 million USD), cabazitaxel (1.5 million USD), darolutamide (1.2 million USD), olaparib (0.9 million USD) and goserelin (0.1 million USD).

**Table 3:** Expenditure with favorable technical notes and projection of all legal demands, according to the CMED list prices (considering PPP conversion)^(14)^

	Unit cost/ptnt	Annual cost/ptnt	Total expenditure (favorable NT)	Projection of annual expenditure
Gosserelin	70,01	840,38	22.696,77	66.520,25
Abiraterone	1.145,67	1.033,85	15.211.434,83	44.620.204,61
Apalutamide	1.771,38	21.238,43	2.060.279,64	6.042.579,32
Enzalutamide	1.744,90	20.936,44	4.564.003,97	13.388.369,23
Darolutamide	184.421,28	22.138,42	420.386,60	1.233.728,80
Cabazitaxel	2.202,89	13.221,22	515.779,90	1.510.090,09
Olaparib	3.258,26	38.747,63	310.099,42	909.013,68
Radium 223	2.114,17	38.043,92	1.064.764,81	3.124.476,21
Total	—	—	24.159.445,94	70.894.982,19

NT: technical notes; CMED: *Câmara de Regulação do Mercado de Medicamentos;* PPP: Purchasing Power Parity.

As depicted in table 4, regarding procedural demands (n=322), the petitions included complementary examinations (n=106) such as biopsies, bone scintigraphy, PET-CT, TC, or MRI, as well as surgical operations (n=105) and radiotherapy (n=15). The most frequently requested surgeries were prostatectomy (n=53), TURP (n=15), and urinary sphincter implantation (n=12). Patients searching for procedures proportionally obtained the second highest number of positive outcomes for their legal actions, preceded only by those who sought bone protective agents.

**Table 4:** Number of cases of lawsuits demanding operations and their outcome

Type of procedure		Requests	Favorable	Favorable%
Complementary exams		106	77	72.6
	Biopsy	30	29	96.7
	Bone scintigraphy	5	3	60.0
	PET-CT	61	38	62.3
	MRI or CT	10	7	70.0
Radiotherapy		15	9	60.0
Surgical procedure		105	79	75.2
	Robotic surgery	17	8	47.1
	Urinay sphincter implant	12	8	66.7
	Orchiectomy	2	2	100.0
	Prostatectomy	53	43	81.1
	Penile prosthesis	6	5	83.3
	TURP	15	13	86.7
Others		96	65	67.7

TURP: transurethral resection of the prostate; MRI: magnetic resonance imaging; CT: computed tomography.

Regarding healthcare products (n=36), the demands included diapers, postsurgical tampons, and dietary supplements. In terms of legal acceptance, most cases were successful in obtaining the desired product, as the requests represented simple, affordable, and basic makes.

It is also relevant to discuss the demographics of requests issued by NATJUS. The distribution of legal proceedings in Brazilian states is outlined in table 5. Demands were most common in the Southern (n=1,325), followed by the Northeastern (n=983), Southeastern (n=441), Central-western (n=359), and Northern (n=27) regions of Brazil (Table 5).

**Table 5:** Distribution of technical notes according to Brazilian Regions

Region	Requests	Favorable	Favorable%
Central-West	359	169	47.1
North	27	16	59.3
Northeast	983	640	65.1
South	1,325	769	58.0
Southeast	441	255	57.8

**Figure 1:**
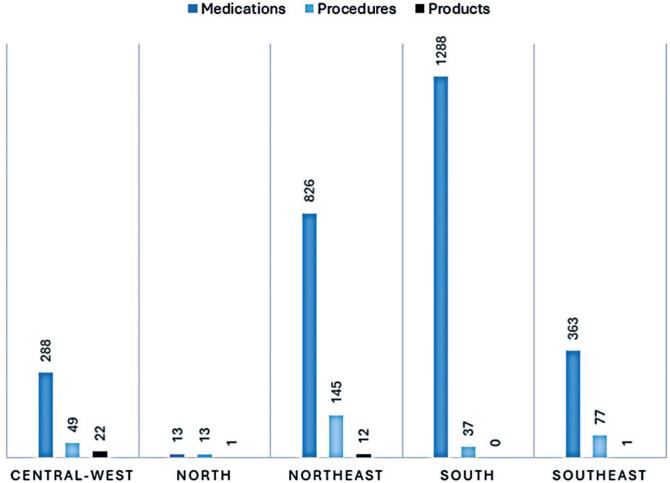
Geographic distribution of lawsuits according to demanded categories in each Brazilian region

Excluding the North, all regions of the country petitioned mostly for medications with a wide advantage margin, considering the number of requests for procedures and products, as shown in figure 1.

A similar distribution can be seen for complementary examinations and surgical procedures, which were mostly requested by Northeastern (n=91) citizens, followed by the Southeast regions (n=77) and Central-west (n=33). The Southeast was also responsible for most of the radiotherapy demands (n= 7) and one of the two requests for robotic surgery. The same was observed in the South, reaffirming the socioeconomic disparities between regions manifested in technical notes.

## DISCUSSION

Despite Brazil's recognition of health as a constitutional right, the Brazilian SUS faces significant challenges in providing adequate universal care, including insufficient funding, unequal distribution of healthcare services across regions, an aging population, incomplete data, and other issues. When the Executive branch cannot ensure proper functioning of the health system, individuals seek recourse through the judicial branch to ensure that their rights are fully upheld.

Access to medical care, especially for oncological patients, is a frequent subject of litigation in Brazilian courts, often resulting in considerable financial burden. Applying this logic to an already expensive disease, such as prostate cancer, can be quite complex, especially considering the rise in both the incidence and prevalence rates of the illness.^(15,16)^ However, disease mortality remains stable, with newer technologies enhancing survival rates.^(17)^ This also implies significant expenses, thereby amplifying the economic strain on the healthcare system.^(18,19)^

Within the period of evaluation (2019-2023), approximately 1,710,170 lawsuits were filed against the Brazilian SUS.^(20)^ The overall expenses related to all these judicial cases have escalated from 6.3 million USD in 2008, to 76.3 million USD in 2014, reaching approximately 180.9 million USD in 2022. As for the technical notes, during the same timeframe, there were 190,675 reported documents. They account for roughly 16.2% of the total lawsuits against the Brazilian SUS. Out of these technical notes, 315,090 aimed for access to medications, with 1.1% (3,535) specifically attributed to prostate cancer. The expenses associated with the technical notes amount to about 41.6 million USD.^(21,22)^

In recent years, federal spending on public health in Brazil has averaged approximately 10.4 billion USD annually. However, there is an estimated yearly deficit of 2 billion USD. Presently, there are 598.8 thousand lawsuits related to health matters concerning medications. Remarkably, more than 90% of these demands involved medications that are not currently covered by the Brazilian SUS.^(23)^ An aggravating factor in this already haunting scenario is the exponential increase in legal demand; for prostate cancer, and the expectation is that these requests might skyrocket in the next few years. This scenario raises the question of system viability.^(24)^ It is vital to promote rational public policies on the incorporation of new technology into the health system to better utilize the financial resources available and, therefore, fulfill the right to access health for more people. To do so, updated data regarding the treatments sought by legal action are crucial, especially considering the contrasting realities in Brazil. Our study revealed several important findings.

For the first time, we were able to better delineate the legal demands for prostate cancer. Access to medications represented 89% of the requests, with androgen pathway inhibitors (abiraterone, enzalutamide, apalutamide, and darolutamide) being so desired that they were responsible for 78.8% of all legal requests associated with prostate cancer diagnoses. Other tools used included examinations (4%), surgery (3%), and radiotherapy (0.4%). This important information should guide public health policies in Brazil.

According to most guidelines, androgen receptor pathway inhibitors are currently important for the treatment of almost all advanced prostate cancers. Several pivotal randomized controlled trials (RCTs) have established the role of Androgen receptor pathway inhibitors in different stages of prostate cancer. Current indications include adjuvant treatment for locally advanced disease, de novo (hormone-sensitive) metastatic disease, nonmetastatic castration-resistant disease, and metastatic castration-resistant disease.^(25)^ These medications are associated with a significant increase in overall survival (OS). A Swedish study evaluating 11,382 men demonstrated that the incorporation of an androgen receptor pathway inhibitor into the treatment of metastatic prostate cancer doubled the 10-year life expectancy from 9 to 18%.^(26)^ It has been more than seven years since RCTs demonstrated that doublet therapy with androgen deprivation therapy and androgen receptor pathway inhibitors increased the OS of metastatic prostate cancer by up to 17 months.^(27- 30)^

Since then, recommendation practices have changed widely worldwide.^(26)^ However, androgen receptor pathway inhibitors are not available to Brazilian patients who rely on the public system. Abiraterone was approved by the CONITEC in 2019 for the treatment of metastatic castration-resistant prostate cancer after chemotherapy. The data on other drugs, apalutamide, enzalutamide, and darolutamide are still unavailable. Since the most common recommendations for androgen receptor pathway inhibitors are currently in a hormone-sensitive setting, there are inconsistencies in clinical indications and availability.. In Brazil, since there are no current recommendations for prostate cancer screening according to health policies, 54.4% of all diagnoses occur in advanced stages, which might skyrocket the requirement for androgen receptor pathway inhibitors in the coming years.^(31)^

**Figure 2:**
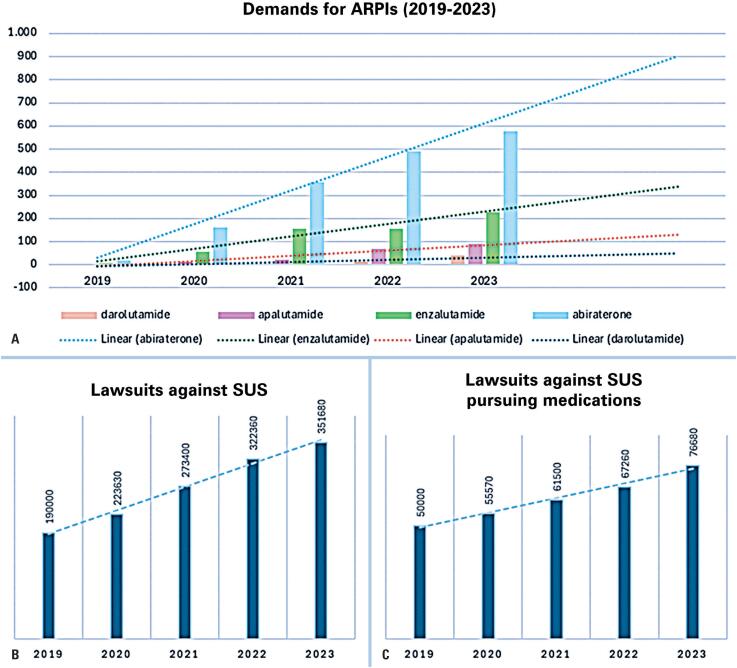
Lawsuits with technical notes against SUS from 2019 to 2023. (A) Lawsuits pursuing androgen receptor pathway inhibitors (abiraterone, enzalutamide; apalutamide or darolutamide); (B) Total number of lawsuits against SUS; (C) Lawsuits pursuing medications

Second, the increase in legal demand over time can be evaluated. We gathered information not only associated with legal demands on the public system, but also with prostate cancer. It is already known that health-related legal demands have increased within the last decade in Brazil.^(32)^ In the last five years, new legal demands against the Brazilian SUS have increased by more than 50%, from 190,630 in 2019 to 351,680 in 2023. The legal demand for medications followed the same trend, increasing from nearly 50,000 in 2019 to more than 76,000 in 2023. During the same period, the demand for androgen receptor pathway inhibitors increased more than 30-fold (Figure 2). Moreover, in 2019 there were 30 demands for abiraterone, apalutamide, enzalutamide, and darolutamide, and in 2023 there were 937 demands with technical notes.

ARPIs: androgen receptor pathway inhibitors.

Third, we estimated expenditures related to prostate cancer demands in the public system. Legal demands for androgen receptor pathway inhibitors are associated with a yearly expenditure of 65.0 million USD. Considering not only androgen receptor pathway inhibitors but also gosserelin, olaparib, and radium 223, the expenditures reached 70.9 million USD/year, which are remarkable findings. For instance, the Federal Government spent 39.9 million USD on chemotherapy and radiotherapy for the treatment of men with prostate cancer in 2023.^(31)^ However, this expenditure was for all men undergoing prostate cancer treatment in the public system in Brazil (71,740 men/year), while 70.9 million USD were spent with an estimated 8,147 legal demands (2,036/year) requiring medications to treat prostate cancer. Expenditures with favorable technical notes associated with androgen receptor pathway inhibitors represented 22.0 million USD, with a projection of 65.3 million USD. If an increment of almost 20% per year is considered, this might become unsustainable for the Brazilian SUS in a short term if nothing changes.^(32)^

It is also important to mention that, despite most of the previously mentioned drugs being approved for use by CONITEC, hardly any of them are included in the list of mandatory distribution drugs known as RENAME. Therefore, by purchasing these drugs only when in demand and in lower quantities, the proportional cost of each medication increases. This clarifies that without rational public policies, only a few health service users will benefit from this investment.

Fourth, we observed regional variations in the health-related legal demands in Brazil. Brazil has five distinctive geographic regions with wide socioeconomic disparities. There are currently almost 600 thousand active legal demands against the Brazilian SUS, representing a rate of 281 demands for every 1,000 inhabitants in Brazil. In the Southern region, the rates are as high as 5.25:1,000 while in the Northern region, legal demands occur at a rate of 0.77:1,000 inhabitants. Almost half of androgen receptor pathway inhibitors were requested in the Southern region (46.7%), where 13% of the Brazilian population resides. In contrast, in the Northern region, there were only 76 (3.1%) demands for androgen receptor pathway inhibitors (7% of the Brazilian population).

Our data demonstrate that, while some patients request basic health products or services (such as diapers or specialized consultations) and relatively affordable treatments, both of which should be readily provided, others seek access to new and expensive technologies. This discrepancy is not only seen among Brazilian regions, with Northern and Northeastern regions taking a bigger tool, given their less favorable socioeconomic development, but also inside the country's states and even cities.

It is also important to note that such regional discrepancies can be partially explained by the difference in population distribution among these regions, with the North sheltering a significantly smaller number of residents. Another aspect to consider is the fewer diagnostics performed in poorer estates given the precarious health services they possess.

Not only should geographic differences be considered in this interpretation but it is also important to consider the impact of socioeconomic status on cancer diagnosis and treatment. One could argue that even the results of the judicialization process might be biased. Because decision-making is based on individual case analysis, the quality of the argument presented to the judge is essential to achieve a positive outcome. Therefore, those with access to lawyers and who are more capable of dealing with the bureaucracy of the judicial branch might be more prone to take legal action and more likely to succeed in their pursuits, since they can afford more experienced and specialized professionals, thus reinforcing the gap in how patients’ health is treated in Brazil. However, this is merely speculation, since there are no available data regarding the number of lawsuits related to personal or family income.

## CONCLUSION

Our study highlights that without prompt, rational, and effective measures, the system is at risk of collapse. In this context, additional research and partnerships between the government, scientific community, judiciary, and patient advocacy groups are imperative. Embracing new technologies is a significant asset to medical care given the absence of approval by RENAME for most of the drugs requested for the treatment of prostate cancer. Overall, there is an urgent need for the Brazilian government to confront this reality and devise and implement viable solutions for the betterment of patient care.
